# 开发和验证一种预后的列线图来指导肺大细胞神经内分泌癌的决策

**DOI:** 10.3779/j.issn.1009-3419.2023.101.21

**Published:** 2023-07-20

**Authors:** Sheng CHEN, Shaoxiang LI, Zipeng WANG, Wenxi ZHANG, Liang ZHOU, Wenjie JIAO

**Affiliations:** 266071 青岛，青岛大学附属医院胸外科; Department of Thoracic Surgery, Affiliated Hospital of Qingdao University, Qingdao 266071, China

**Keywords:** 肺肿瘤, 肺大细胞神经内分泌癌, 风险因素, 列线图, Lung neoplasms, Lung large cell neuroendocrine carcinoma, Risk factors, Nomogram

## Abstract

**背景与目的** 肺大细胞神经内分泌癌（lung large cell neuroendocrine carcinoma, LCNEC）是一种罕见且预后极差的肺恶性肿瘤。目前对LCNEC的研究大多基于回顾性研究，缺乏现实世界的验证。本研究旨在识别独立的危险因素，建立并验证LCNEC预后的预测模型。**方法** 从2010至2015年监测、流行病学和结果数据库（Surveillance, Epidemiology, and End Results, SEER）和我科2016至2020年住院患者中提取患者资料。采用Kaplan-Meier法评估总生存期（overall survival, OS）。OS被定义为患者从确诊到死亡或最后一次随访时间。单因素Cox回归分析和多因素Cox回归分析确定显著的预后因素。从而构建预测LCNEC预后的Nomogram。**结果** 共纳入1892例LCNEC患者，分为训练队列（n=1288）和两个验证队列（n=552, n=52）。单因素Cox回归分析显示年龄、性别、肿瘤原发部位、偏侧、肿瘤原发灶-淋巴结-转移（tumor-node-metastasis, TNM）各分期、手术、放疗可影响LCNEC的预后（P<0.05），多因素Cox分析显示年龄、性别、肿瘤原发部位、T分期、N分期、M分期、手术、放疗是LCNEC患者预后的独立危险因素（P<0.05）。校准曲线和一致性指数（内部：0.744±0.015；外部：0.763±0.020、0.832±0.055）显示模型预测性能良好。**结论** 年龄≥65岁、男性、TNM分期晚、未经过手术或放疗的患者预后不佳。列线图可以对患者的个性化临床决策提供一定的参考。

## Abstract (en)

**Background and objective** Lung large cell neuroendocrine carcinoma (LCNEC) is a rare and highly malignant lung tumor with a poor prognosis. Currently, most research on LCNEC is based on retrospective studies and lacks validation in the real world. The study aims to identify independent risk factors and establish and validate a predictive model for the prognosis of LCNEC. **Methods** Patient data were extracted from Surveillance, Epidemiology, and End Results (SEER) and our department's hospitalization records from 2010 to 2015 and 2016 to 2020, respectively. Kaplan-Meier analysis was used to evaluate overall survival (OS). OS is defined as the time from diagnosis to death or last follow-up for a patient. Univariate and multivariate Cox regression analyses were performed to identify significant prognostic factors and construct a Nomogram for predicting the prognosis of LCNEC. **Results** In total, 1892 LCNEC patients were included and divided into a training cohort (n=1288) and two validation cohorts (n=552, n=52). Univariate Cox regression analysis showed that age, gender, primary tumor site, laterality, T stage, N stage, M stage, surgery, and radiotherapy were factors that could affect the prognosis of LCNEC patients (P<0.05). Multivariate Cox analysis indicated that age, gender, primary tumor site, T stage, N stage, M stage, surgery, and radiotherapy were independent risk factors for the prognosis of LCNEC patients (P<0.05). Calibration curves and the concordance index (internal: 0.744±0.015; external: 0.763±0.020, 0.832±0.055) demonstrated good predictive performance of the model. **Conclusion** Patients aged ≥65 years, male, with advanced tumor-node-metastasis (TNM) staging, and who have not undergone surgery or radiotherapy have a poor prognosis. Nomogram can provide a certain reference for personalized clinical decision-making for patients.

癌症是全球最为主要的公共健康问题，癌症相关死亡原因主要以肺恶性肿瘤居多（占癌症死亡总数的18%）^[1]^。肺大细胞神经内分泌癌（lung large cell neuroendocrine carcinoma, LCNEC）是一种罕见的肺癌亚型，约占肺恶性肿瘤的3%^[2]^。来自美国和荷兰的统计数据^[3]^显示，LCNEC的发病率正逐年上升。与此同时，LCNEC的预后较差，与小细胞肺癌相似，伴有进展快和易转移的临床特点^[4]^。有研究^[5]^显示，55.25%的患者在确诊时已伴有远处转移，肝、骨、脑、肺是最常见的转移部位。值得思考的是，一旦发生远处转移，LCNEC的1年生存率仅为8%-17%，中位生存期仅为4-8个月^[6]^。

目前LCNEC尚未有最佳的综合治疗方案，其中手术治疗是治疗早期局限性LCNEC的唯一有效手段^[7]^。由于其分子层面的高度异质性，LCNEC的治疗尚存在争议^[8]^。虽然已有研究对LCNEC的预后相关因素进行了分析，但大多缺乏现实世界的验证^[9]^。因此，迫切需要建立起符合现实情形的LCNEC预后模型，以促进临床危险筛查和生存结局评估。

本文基于监测、流行病学和最终结果数据库（Surveillance, Epidemiology, and End Results, SEER），分析确定与LCNEC生存相关的潜在危险因素，并开发和验证一个Nomogram生存预测图。

## 1 资料与方法

### 1.1 数据来源

SEER数据库是世界上最大的癌症公开数据库之一，覆盖了美国28%的癌症患者^[10]^。本文数据来源为“Incidence-SEER Research Plus Data, 18 registries, Nov 2020 Sub (2000-2018)"。使用SEER*Stat软件8.4.0版本（https://seer.cancer.gov/seerstat/）从SEER数据库（http://seer.cancer.gov/）中提取2010至2015年诊断为LCNEC的患者数据。将ICD-O-3编码为8013/3的肺癌患者进行分析。纳入标准：（1）患者相关生存资料完整；（2）经过病理学或免疫组化确诊；（3）手术、放化疗等治疗信息明确。排除标准：（1）生存资料缺失；（2）肿瘤原发灶-淋巴结-转移（tumor-node-metastasis, TNM）分期不明确；（3）转移情况未知；（4）肿瘤原始部位和分级程度丢失。与此同时，我们还收集了2016至2020年在我院诊治的LCNEC患者的数据。由于SEER的公开性，且我们已经获得访问和使用该数据库的授权许可，因而本研究并不需要相关伦理委员会的批准。此外，涉及到本院患者的研究，已获得青岛大学附属医院伦理委员会的批准（批准号：QYFY WZLL 27998），并无需获得知情同意。

从SEER数据库中提取出LCNEC患者的以下信息：患者的人口统计学数据如诊断年龄、性别、种族、肿瘤特征（部位、偏侧、组织学分级、TNM分期、治疗史（手术、放疗、化疗）及生存资料[存活状态和总生存期（overall survival, OS）]。OS被定义为患者从确诊到死亡或最后一次随访的时间。特别提醒的是，肿瘤分期是根据第七版美国国家癌症联合委员会的TNM分期来制定的。符合纳排标准的患者按照7:3随机分为训练队列和验证队列。70% SEER来源的患者作为训练队列数据构建Nomogram，30% SEER来源的患者和我们数据库来源的患者分为两个验证队列，作为Nomogram的外部验证。

### 1.2 数据分析

患者的临床基线特征用计数和百分比来表示。使用Kaplan-Meier法绘制累计生存曲线，比较采用Log-rank检验，使用Cox比例风险回归模型评估临床病理变量与生存时间之间的关系。通过单变量Cox回归分析，筛选出具有统计学意义的临床病理变量。将得到的临床病理变量纳入到多因素Cox回归分析，确定独立的预后因素。从而建立起关于LCNEC生存相关的预后Nomogram图。采用一致性指数（concordance index, C-index）、校准曲线评估和验证Nomogram模型的性能。30%SEER来源的患者和我们数据库来源的患者作为两个外部验证，进一步验证列线图的预测能力。所有检验为双侧检验，P<0.05为具有统计学差异。本文中出现的统计分析均由R软件4.2.3（R Foundation for Statistical Computing, Vienna, Austria）进行。

## 2 结果

### 2.1 LCNEC的一般特征

从SEER数据库中初步识别出31,064例肺癌患者，纳入1840例符合纳排标准的LCNEC患者。此外，收集青岛大学附属医院诊治的LCNEC患者52例。最后，将SEER数据库中的1288例LCNEC患者随机分配到训练队列中，其余的552例LCNEC患者和青岛大学附属医院收治的52例LCNEC患者纳入到验证队列。详细的筛选过程如图1的流程图所示，人口学信息和肿瘤特征见表1。

**图1:**
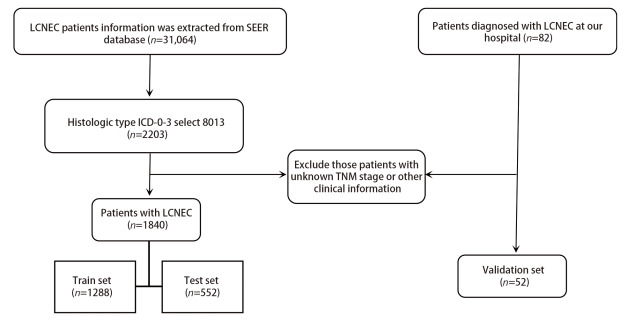
本研究选择过程的流程图

**表1:** 1892例LCNEC患者的一般资料

Variables		SEER database (n=1840),n (%)	Training cohort (70% SEER database,n=1288), n(%)	Validation cohorts, n (%)	
				30% SEER database (n=552)	Database of our department(n=52)
Age (yr)	<65	757 (41.1)	511 (39.7)	246 (44.6)	34 (65.4)
	≥65	1083 (58.9)	777 (60.3)	306 (55.4)	18 (34.6)
Race	White	1537 (83.5)	1087 (84.4)	450 (81.5)	0 (0.0)
	Black	231 (12.6)	133 (10.3)	98 (17.8)	0 (0.0)
	Others	72 (3.9)	68 (5.3)	4 (0.7)	52 (100.0)
Gender	Female	841 (45.7)	622 (48.3)	219 (39.7)	7 (13.5)
	Male	999 (54.3)	666 (51.7)	333 (60.3)	45 (86.5)
Primary site	Upper lobe of lung	1092 (59.3)	759 (58.9)	333 (60.3)	29 (55.8)
	Middle lobe of lung	77 (4.2)	52 (4.0)	25 (4.5)	0 (0.0)
	Lower lobe of lung	493 (26.8)	349 (27.1)	144 (26.1)	23 (44.2)
	Overlapped lobes of lung	25 (1.4)	19 (1.5)	6 (1.1)	0 (0.0)
	Unspecified	153 (8.3)	109 (8.5)	44 (8.0)	0 (0.0)
Grade	I	8 (0.4)	4 (0.3)	4 (0.7)	0 (0.0)
	II	20 (1.1)	12 (0.9)	8 (1.4)	0 (0.0)
	III	695 (37.8)	488 (37.9)	207 (37.5)	25 (48.1)
	IV	218 (11.8)	162 (12.6)	56 (10.1)	27 (51.9)
	Unknown	899 (48.9)	622 (48.3)	277 (50.2)	0 (0.0)
Laterality	Right	1070 (58.2)	749 (58.2)	321 (58.2)	31 (59.6)
	Left	739 (40.2)	514 (39.9)	225 (40.8)	21 (40.4)
	Bilateral	31 (1.7)	25 (1.9)	6 (1.1)	0 (0.0)
T stage	T1	491 (26.7)	349 (27.1)	142 (25.7)	23 (44.2)
	T2	571 (31.0)	389 (30.2)	182 (33.0)	13 (25.0)
	T3	407 (22.1)	279 (21.7)	128 (23.2)	9 (17.3)
	T4	371 (20.2)	271 (21.0)	100 (18.1)	7 (13.5)
N stage	N0	806 (43.8)	580 (45.0)	226 (40.9)	23 (44.2)
	N1	183 (9.9)	125 (9.7)	58 (10.5)	10 (19.2)
	N2	613 (33.3)	429 (33.3)	184 (33.3)	10 (19.2)
	N3	238 (12.9)	154 (12.0)	84 (15.2)	9 (17.3)
M stage	M0	989 (53.8)	689 (53.5)	300 (54.3)	24 (46.2)
	M1	851 (46.3)	599 (46.5)	252 (45.7)	28 (53.8)
Surgery	Yes	704 (38.3)	501 (38.9)	203 (36.8)	34 (65.4)
	No	1136 (61.7)	787 (61.1)	349 (63.2)	18 (34.6)
Radiotherapy	Yes	723 (39.3)	486 (37.7)	237 (42.9)	16 (30.8)
	No/Unknown	1117 (60.7)	802 (62.3)	315 (57.1)	36 (69.2)
Chemotherapy	Yes	954 (51.8)	653 (50.7)	301 (54.5)	37 (71.2)
	No/Unknown	886 (48.2)	635 (49.3)	251 (45.5)	15 (28.8)

LCNEC: lung large cell neuroendocrine carcinoma; SEER: Surveillance, Epidemiology, and End Results.

2010至2016年，SEER数据库中共有1840例患者诊断为LCNEC，其中65岁以下的患者占41.1%（n=757），65岁及以上的患者占58.9%（n=1083）。男性患者占54.3%（n=999）。白种人的发病率最高为83.5%（n=1537），其次为黑种人（n=231, 12.6%）和其他种族（n=72, 3.9%）。上叶为最常见的肿瘤原发部位，占59.3%（n=1092）。低分化LCNEC占37.8%（n=695），其次是未分化LCNEC（n=218, 11.8%），高分化和中分化LCNEC占比较少，分别为0.4%（n=8）和1.1%（n=20）。右肺LCNEC的发生率较左肺高（58.2% vs 40.2%）。手术、放疗、化疗分别为704例（38.3%）、723例（39.3%）和954例（51.8%）。

### 2.2 LCNEC的生存分析

整体LCNEC的中位生存时间为11个月（95%CI: 10.0-12.0）（图2）。一旦发生远处转移，LCNEC的预后极差（图3）。肺转移、脑转移、骨转移、肝转移和多发转移患者的中位生存期分别为7个月（95%CI: 5.25-8.75）、5个月（95%CI: 3.64-6.36）、6个月（95%CI: 3.14-8.86）、3个月（95%CI: 1.54-4.46）和4个月（95%CI: 3.35-4.65）。对训练队列进行单因素分析，确定LCNEC相关的预后因素。最终，确定9个变量，包括年龄、性别、肿瘤原发部位、偏侧、T分期、N分期、M分期、手术、放疗（P<0.05）（表2）。接着使用多因素分析9个潜在的预测预后因子，结果显示，年龄、性别、肿瘤原发部位、T分期、N分期、M分期、手术、放疗作为OS的预测因子具有统计学意义（图4）。

**图2:**
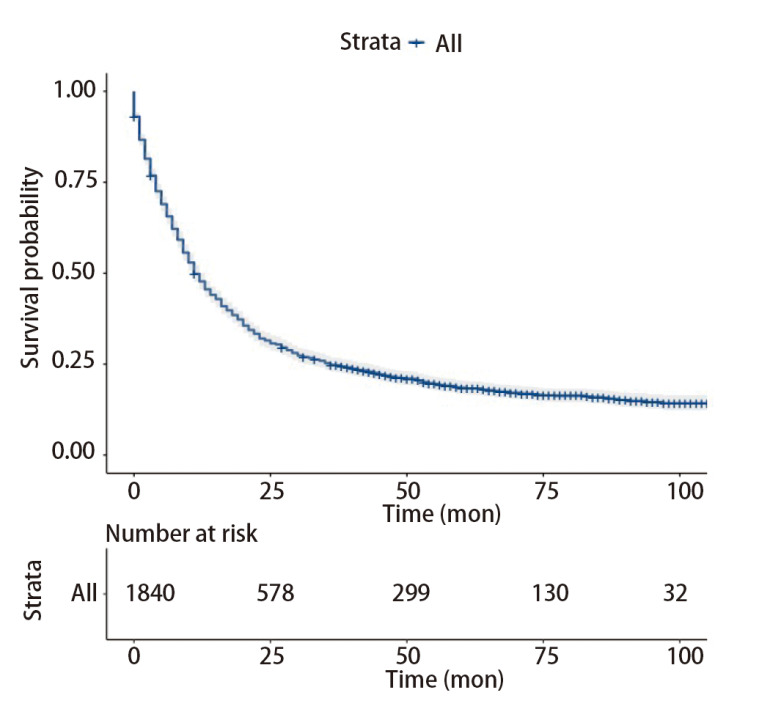
总体生存率的Kaplan-Meier曲线和风险表

**图3:**
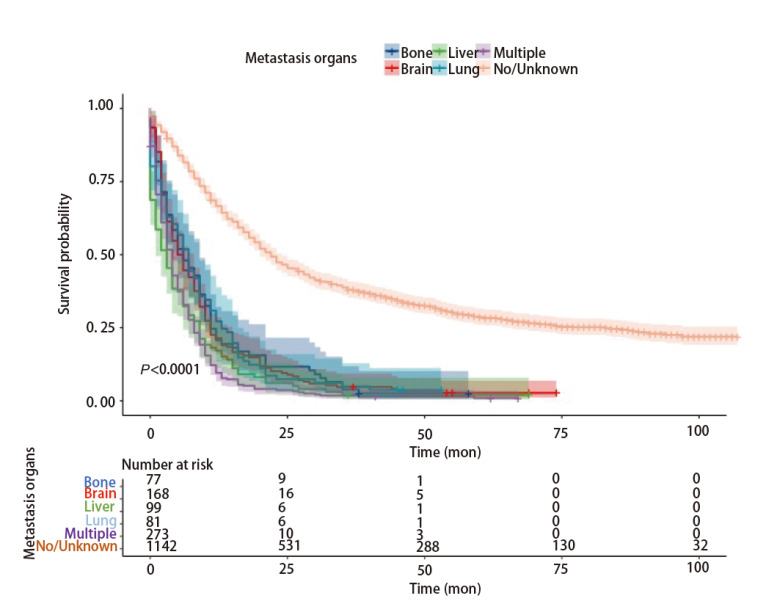
伴有远处转移的生存率的Kaplan-Meier曲线和风险表

**表2:** LCNEC患者的Cox比例风险回归分析

Variables		Univariate analysis				Multivariate analysis		
		HR	95%CI	P		HR	95%CI	P
Age (yr)	<65			Reference				
	≥65	1.17	1.03-1.33	0.013		1.29	1.14-1.47	<0.001
Race	Black			Reference				
	White	1.08	0.89-1.33	0.430				
	Others	1.28	0.94-1.76	0.120				
Gender	Female			Reference				
	Male	1.26	1.12-1.42	<0.001		1.20	1.06-1.36	0.004
Primary site	Upper lobe of lung			Reference				
	Middle lobe of lung	1.18	0.87-1.60	0.287		1.08	0.79-1.48	0.617
	Lower lobe of lung	1.11	0.97-1.28	0.139		1.06	0.92-1.22	0.419
	Overlapped lobes of lung	1.94	1.20-3.14	0.007		1.75	1.08-2.85	0.024
	Unspecified	2.09	1.69-2.58	<0.001		1.07	0.84-1.38	0.571
Grade	I			Reference				
	II	0.65	0.16-2.59	0.539				
	III	1.23	0.40-3.84	0.719				
	IV	1.44	0.46-4.52	0.533				
	Unknown	2.38	0.77 7.41	0.134				
Laterality	Left			Reference				
	Right	1.06	0.94-1.21	0.336		1.00	0.88-1.13	0.942
	Bilateral	2.35	1.56-3.56	<0.001		1.02	0.64-1.64	0.929
T stage	T1			Reference				
	T2	1.66	1.40-1.96	<0.001		1.21	1.02-1.45	0.031
	T3	2.20	1.84-2.64	<0.001		1.30	1.07-1.58	0.009
	T4	3.24	2.71-3.89	<0.001		1.37	1.11-1.69	0.003
N stage	N0			Reference				
	N1	1.81	1.46-2.24	<0.001		1.50	1.21-1.87	<0.001
	N2	2.68	2.32-3.09	<0.001		1.36	1.15-1.60	<0.001
	N3	3.34	2.75-4.06	<0.001		1.55	1.24-1.93	<0.001
M stage	M0			Reference				
	M1	4.15	3.64-4.73	<0.001		2.34	1.99-2.76	<0.001
Surgery	No			Reference				
	Yes	0.27	0.23-0.31	<0.001		0.48	0.39-0.59	<0.001
Radiotherapy	No/Unknown			Reference				
	Yes	1.36	1.20-1.54	<0.001		0.77	0.67-0.88	<0.001
Chemotherapy	No/Unknown			Reference				
	Yes	1.03	0.91-1.16	0.628				

HR: hazard ratio.

**图4:**
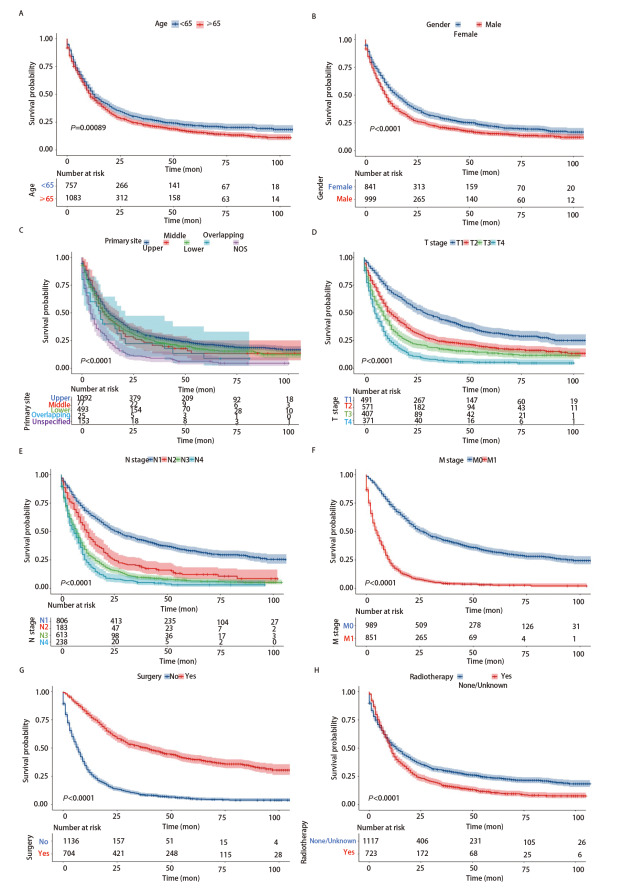
采用Kaplan-Meier法，根据年龄（A）、性别（B）、肿瘤原发部位（C）、T分期（D）、N分期（E）、M分期（F）、手术（G）、放疗（H）预测患者的OS。

### 2.3 列线图的开发和验证

利用年龄、性别、肿瘤原发部位、T分期、N分期、M分期、手术史和放疗史这8个独立危险因素制作预测LCNEC预后的列线图（图5）。独立危险因素累计的总分数对应着1、3和5年的预测概率。使用校准曲线评估模型预测的生存率与实际的差距，一般来说，在一个校准良好的模型中，预测应该落在45°的对角线上。预测1、3、5年的OS的C-index为0.744±0.015，表明该模型与实际观察结果差距不大。

**图5:**
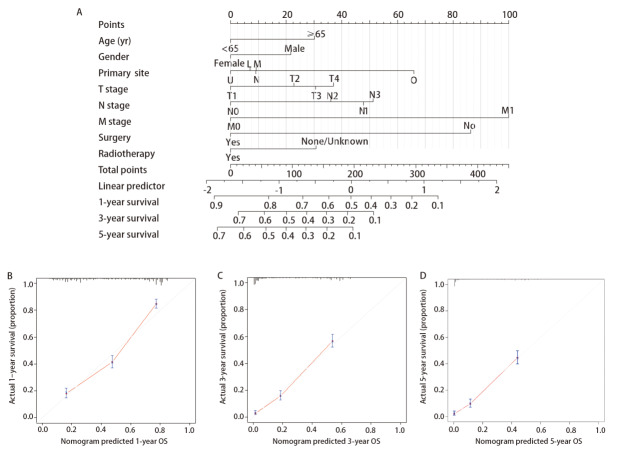
预测列线图及其验证的校准曲线。训练队列中LCNEC患者1、3和5年OS预测的Nomogram预测图（A）；预测训练队列中LCNEC患者1（B）、3（C）、5（D）年OS率的Nomogram校准曲线。

使用30% SEER数据库和青岛大学附属医院的数据库对该Nomogram进行外部验证，模型预测的生存率和实际生存率相比大致符合，证明该模型的性能尚可。两个验证集的C-index分别是0.763±0.020（30% SEER数据库）和0.832±0.055（本院数据库）。校准图如图所示，此外因为本院数据库中5年生存期患者的数据较少，所以无法绘制5年Nomogram的校准曲线（图6）。

**图6:**
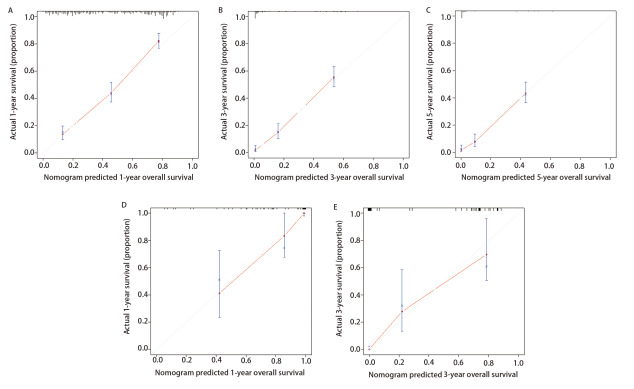
列线图外部验证的校准曲线。预测SEER数据库验证队列中LCNEC患者1（A）、3（B）、5（C）年OS率的列线图校准曲线；预测我院数据库验证队列中LCNEC患者1（D）、3（E）年OS率的列线图校准曲线。

## 3 讨论

在本研究中，我们团队分析了LCNEC患者预后的危险因素。通过对1892例患者临床资料的分析，显示年龄、性别、肿瘤原发部位、T分期、N分期、M分期、手术、放疗是独立的预后危险因素，从而绘制出可以直观预测患者生存期的Nomogram图。

在我们的研究中，SEER数据库登记的患者诊断时的年龄分布（≥65岁，58.9%）与本院登记的患者诊断年龄分布（≥65岁，34.6%）不同。考虑是近年肺癌早期筛查推广的原因。既往的一项小型回顾性研究^[11]^显示，LCNEC常见于有重度吸烟史的老年男性。与此同时，我们观察到男性LCNEC群体比女性LCNEC群体的预后更差，这可能与男性吸烟史有关。我们的研究还表明高年龄段（≥65岁）的预后差，这可能与老年患者的体能状态差和合并基础疾病有关^[12]^。T分期、N分期、M分期、手术史等变量均已有相关研究^[13⇓-15]^证实为显著的LCNEC的预后不良因素。本研究发现肿瘤的原发部位与LCNEC预后相关。Lee等^[16]^分析显示上叶肺癌有更好的临床结局。目前关于该现象尚无明确的解释，最常见的理论是下叶肿瘤确定N分期或T分期较为困难，一些位于胸膜附近或气道附近的肿瘤，手术后的病理分期可能需要上调^[17]^。此外，下肺肿瘤更容易扩散到隆突下、食管旁或下肺韧带淋巴结^[18]^。列线图显示LCNEC患者重叠病变预后最差，值得以后进一步讨论。

LCNEC是一种较为罕见的肺恶性肿瘤，尽管部分患者接受了积极的抗癌治疗，但效果并不理想。有研究^[19]^显示LCNEC患者的5年生存率仅为15%-20%。当下关于LCNEC的治疗方法仍然存在争议^[20]^。手术和放化疗是临床上最常见的LCNEC治疗手段。美国国立综合癌症网络指南建议对I-IIB期的LCNEC患者进行手术治疗，对局部晚期（III期）患者采用化放疗+手术的综合治疗，不建议对IV期患者行手术治疗^[21]^。可见手术治疗是可切除LCNEC的基石。Gu等^[22]^的研究显示，接受手术的I、II和III期LCNEC患者的预后明显优于非手术患者（P<0.05）。值得讨论的是，在本研究中化疗在延长LCNEC的OS中显示出有限的效果（P>0.05）。这可能与罹患LCNEC患者的年龄较大、身体状况差、无法耐受化疗有关。在一项回顾性研究^[23]^中确定了辅助化疗对患者生存的裨益，肿瘤直径>3 cm且在手术后3-6个月内进行化疗能获得更好的OS。但肿瘤直径<2 cm且在手术6个月开始化疗获益并不明显。Deng等^[15]^也证实了化疗对IB-IIIA期患者的积极作用^[24]^。此外，LCNEC的标准化疗方案仍存在争议，使用不同的化疗方案可能导致不同的临床结局。我们期待前瞻性临床研究能够证实化疗在临床的作用，并对治疗决策提供指导。在本研究中放疗对改善LCNEC患者OS有着积极的影响。Cao等^[25]^研究证实，放疗可以为II和III期的LCNEC患者提供显著的生存获益，与无RT组相比，RT组的1、3和5年OS率更高（分别为41.2%、14.8%和8.8%，vs 30.9%、11.2%和6.1%，P<0.001）。因此，我们建议，对于I、II和III期LCNEC患者，可以采用放化疗联合手术切除的方式，以期获得更好的临床结果。

本研究存在一定的局限性。首先，SEER数据库没有提供放化疗的具体信息，包括用药方案及辐射范围；其次，纳入的临床特征有限。例如体能状态评分、吸烟史、基因突变状态等都是与肿瘤预后相关的重要因素，这些因素均未纳入本研究中；最后，回顾性研究的选择偏倚无法避免。未来有必要进行大型随机临床试验来验证本文的结果。

综上所述，我们的研究结果提示，年龄、性别、肿瘤原发部位、T分期、N分期、M分期、手术、放疗是影响患者生存率的主要因素。此外，本研究成功构建出LCNEC预后的列线图，可以根据临床病理特征预测每一例LCNEC患者的生存期，具有一定的参考价值。

**Competing interests**

The authors declare that they have no competing interests.

**Author contributions**

Chen S designed the study. Chen S and Wang ZP analyzed the data. Zhang WX and Zhou L contributed analysis tools. Chen S, Li SX, Wang ZP and Zhang WX provided critical inputs on design, analysis, and interpretation of the study. All the authors had access to the data. All authors read and approved the final manuscript as submitted.
